# Spongiform Encephalopathy in a Miniature Zebu

**DOI:** 10.3201/eid1212.060750

**Published:** 2006-12

**Authors:** Torsten Seuberlich, Catherine Botteron, Christian Wenker, Valeria Café-Marçal, Anna Oevermann, Bianca Haase, Tosso Leeb, Dagmar Heim, Andreas Zurbriggen

**Affiliations:** * University of Berne, Berne, Switzerland;; † Zoo Basel, Basel, Switzerland;; ‡ Federal Veterinary Office, Berne, Switzerland

**Keywords:** spongiform encephalopathy, BSE, cattle, prion, prion protein, Switzerland, zebu, scrapie, dispatch

## Abstract

The first case of spongiform encephalopathy in a zebu (*Bos indicus*) was identified in a zoo in Switzerland. Although histopathologic and immunohistochemical analyses of the central nervous system indicated a diagnosis of bovine spongiform encephalopathy (BSE), molecular typing showed some features different from those of BSE in cattle (*B. taurus*).

Spongiform encephalopathies (SEs) are transmissible neurodegenerative diseases characterized by spongiform lesions and deposition of partially proteinase K–resistant prion protein (PrP^sc^), a misfolded isoform of the normal host-encoded cellular prion protein (PrP^c^), in the central nervous system (CNS). The oldest known SE is scrapie, which occurs naturally in sheep and goats. Since the onset of the bovine spongiform encephalopathy (BSE) epidemic in British cattle (Bos taurus) in 1986, novel SEs emerged in other animal species including domestic cats (*1*), a goat (*2*), primates (*3*), and several members of the families Bovidae and Felidae in zoos (*4**,**5*). Experimental and epidemiologic evidence indicate that these animals were infected by ingesting BSE-infected carcasses or meat and bone meal.

Zebus (B. indicus) belong to the family Bovidae. In Asia they are raised mainly as productive livestock, but in Europe they live primarily in zoos. We describe clinical, pathologic, and molecular features of the first case of SE in a zebu and address the question whether this animal was infected with the BSE agent.

## The Study

**Figure 1:**
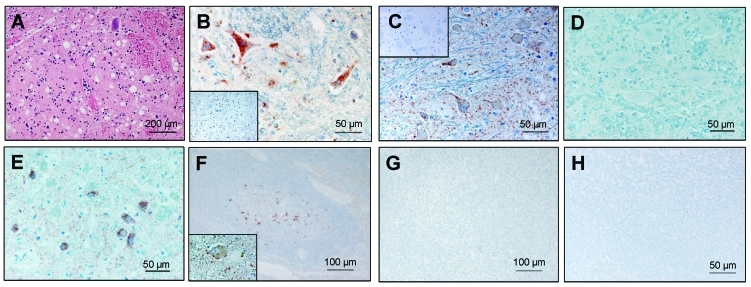
Histopathologic and immunohistochemical analyses. A) Spongiform lesions; B) partially proteinase K–resistant prion protein (PrP^sc^) deposits detected by immunohistochemistry (monoclonal antibodies [MAb] F99/97.6.1 diluted 1:500) in the nucleus of the solitary tract (STN) in the zebu under investigation. C–E) Comparative immunohistochemistry with MAb P4 (1:800) in the olivary nuclei of the zebu (C), a bovine spongiform encephalopathy (BSE)-positive cow (D), and a scrapie-positive sheep (E). Insets show control tissue slides of BSE-negative cattle. F–H) Immunohistochemistry for PrP^sc^ in lymphoid tissue of the zebu (H, mediastinal lymph node), and a BSE-negative cow (G, mandibular lymph node) with MAb L42 (R-biopharm, 1:800). A retropharyngeal lymph node of a scrapie-affected sheep (F) and a brainstem tissue slide of the zebu (F, inset) served as positive controls. Pretreatment of the tissue slides comprised a proteinase K–digestion step (5 μg/mL, 15 min, 37°C).

In 2004, a 19-year-old miniature zebu in a zoo in Basel, Switzerland, fell during mating, after which it had abnormal gait and posture. After 6 weeks it started to bump into obstacles and showed anxiety and loss of proprioceptive control. Because of its old age and the progressive course of the disease, the animal was euthanized, and multiple organs were examined postmortem (Table 1). Histopathologic examination showed severe spongiform changes and a moderate gliosis in the brainstem (Figure 1A, nucleus of the solitary tract), and many other CNS structures. Immunohistochemical analysis (*6*), which used the monoclonal antibodies (MAbs) F99/97.6.1 (VMRD, Pullman, WA, USA) and P4 (R-biopharm, Darmstadt, Germany), identified a marked deposition of PrP^sc^ in the neuropil (granular type) and the neurons (Figure 1B and 1C). The cerebral cortex contained a moderately increased number of Alzheimer type II cells. Numerous nonnervous tissues, including the lymphoreticular system (Figure 1H), were analyzed by immunohistochemical techniques for the presence of PrP^sc^, but none was found. Taken together, these findings led to the diagnosis of a severe SE in combination with a mild metabolic encephalopathy.

**Table 1:** Pathologic findings in a zebu with spongiform encephalopathy, Switzerland, 2004

Site	Finding
Vertebral column	Severe degeneration of intervertebral discs with ankylosing spondylarthrosis
Joints of extremities	Degenerative joint disease
Liver	Biliary cysts (affecting 60% of the liver)
Kidney	Tubular cysts; mild interstitial nephritis with glomerulosclerosis and tubular atrophy
Urinary bladder	Multiple papillomas
Abdominal cavity	Multiple foci of fat necrosis
Cardiovascular system	Mild coronary arteriosclerosis; mild valvular endocardiosis (mitral valve)
Mediastinal lymph nodes	Focal metastatic neuroendocrine tumor (origin unknown)
Central nervous system	Spongiform encephalopathy; metabolic encephalopathy (hepatoencephalopathy)

**Figure 2:**
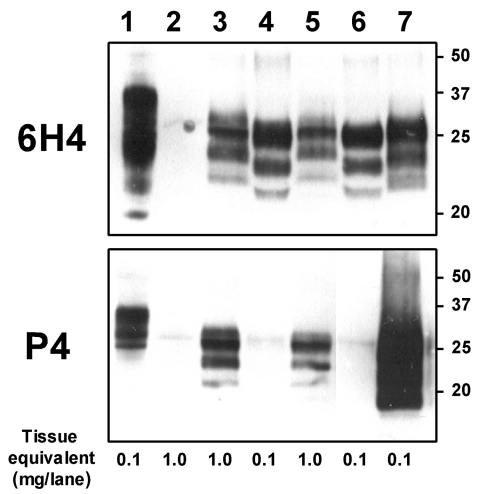
Molecular analyses of the zebu under investigation. Western immunoblot with monoclonal antibodies (MAbs) 6H4 (upper panel) and P4 (lower panel) after limited proteinase K digestion (100 μg/mL, 40 min, 48°C) of 10% brainstem (lanes 3 and 4) and thalamus (lanes 5, 6, and 7) tissue homogenates of the zebu (lanes 3 and 5), a cow with bovine spongiform encephalopathy (lanes 4 and 6), and a sheep with scrapie (lane 7). An undigested cattle brainstem tissue homogenate (lane 1) and a cerebrum tissue homogenate of a spongiform-encephalopathy-negative zebu (lane 2) were included as controls. All samples were processed equally as described by Stack et al. (7), and the membranes were exposed in parallel on the same photographic film. Molecular mass standards in kilodaltons are indicated on the right; tissue mass equivalents, at the bottom.

To assess the possibility that this animal was infected with the BSE agent, we compared the distribution of the SE-related histopathologic lesions and the PrP^sc^ deposits in different brain structures of the zebu to those in the brain of a Swiss BSE-affected cow. In both animals, spongiform lesions were similarly distributed throughout the brain, but overall the lesions in the zebu were more severe than those in the cow (Table 2). The depositions of PrP^sc^ in these structures, as determined by immunohistochemical analysis with MAb F99/97.6.1 and different commercial BSE screening tests (Check Western, Prionics, Zurich, Switzerland; TeSeE, Bio-Rad, Marnes-la-Coquette, France), were well associated with the histopathologic lesions in both animals (data not shown). In comparative Western immunoblot (WB) analysis that used MAb 6H4 (Prionics), the zebu CNS samples (Figure 2, lanes 3 and 5) showed a characteristic 3-band pattern representing un-, mono- and diglycosylated moieties of the proteinase K–resistant PrP^sc^ fragment. In the zebu these 3 bands clearly showed a migration pattern at a higher molecular mass than that of BSE in the cow (Figure 2, lanes 4 and 6) but similar to a sample from a sheep with scrapie (Figure 2, lane 7). When samples of the same animals were analyzed by WB (Figure 2) and immunohistochemical analysis (Figure 1C–E) with P4, an MAb used to discriminate between BSE and scrapie in sheep (*7*), PrP^sc^ was detectable in samples from the sheep with scrapie and the zebu under investigation but not in the cow with BSE. Sequencing of the open reading frame of the Prnp gene of the zebu confirmed that the encoded PrP protein was identical to the B. taurus PrP amino acid sequence (as translated from GenBank accession no. AJ298878).

**Table 2:** Histopathologic lesions in brain of zebu with spongiform encephalopathy and cow with bovine spongiform encephalopathy, Switzerland, 2004*

Site	Cow	Zebu
Brainstem		
Dorsal motor nucleus of vagus nerve	+	++
Nucleus of spinal tract of trigeminal nerve	++	+++
Nucleus of hypoglossal nerve	+	++
Reticular formation	++	++
Nucleus of solitary tract	++	+++
Vestibular nucli	++	+ to ++
Olivary nuclei	++	++
Cerebellar cortex	–	+
Midbrain (*substantia grisea centralis*)	+ to ++	++ to +++
Thalamus	++	++ to +++
Hippocampus	n.d.	+
Basal nuclei (*pallidum*)	+	+
Cerebral cortex	+	++

*–, absent; +, mild; ++, moderate; +++ severe; n.d., not done.

## Conclusions

In 1990, the first case of BSE in cattle in Switzerland was diagnosed; since then, authorities have banned meat and bone meal in ruminant feed in Switzerland. The zebu was born in 1985 and until 1990 ate commercial pellets containing meat and bone meal. Consequently, it might have been exposed to the BSE agent at <5 years of age.

The clinical signs of the zebu were specific for an SE but could have been explained partially by other pathologic findings, e.g., the degenerative lesions of the spine and the metabolic encephalopathy (Table 1). However, prominent spongiform changes and marked depositions of PrP^sc^ in the brain confirmed the clinical suspicion of an SE. The distribution and type of the lesions (Table 2) and PrP^sc^ deposits in the brain of the zebu were very similar to those in the brain of the Swiss BSE-affected cow and to findings that have been described previously for BSE in cattle in Switzerland (*8**,**9*) and elsewhere (*10**–**12*).

In contrast, molecular analysis of PrP^sc^ clearly showed a difference between the zebu and the BSE cow regarding 1) the apparent molecular mass of the PK-resistant fragment of PrP^sc^ on WB analysis and 2) the immunoreactivity with MAb P4 on WB and immunohistochemical analyses. Both observations can be explained by extended proteinase K cleavage at the N terminus of PrP^sc^ in cattle compared with the zebu, resulting in removal of the P4 epitope (*7*). Recently, very similar molecular findings were reported from France (*13*) in 3 exceptionally old (8, 10, and 11 years) cattle. These animals had an atypical PrP^sc^ WB profile, different from that traditionally seen in cattle with BSE but indistinguishable from those in sheep with natural scrapie and cattle with experimental scrapie. This molecular phenotype was retained after transmission of the disease to C57BL/6 mice (*14*). The authors speculated that their findings may reflect either an infection with another type of infectious agent distinct from BSE, e.g., scrapie, or a sporadic form of SE in cattle. For the zebu, the latter hypothesis is supported by the observation that the molecular features of PrP^sc^ were similar to the ones observed in type 1 sporadic Creutzfeldt-Jakob disease (*15*), an SE in humans. On the other hand, consistent with the findings on WB, MAb P4 readily detected PrP^sc^ by immunohistochemical analyses of the CNS of the zebu and in sheep with scrapie but not in bovine BSE under the conditions used. Extracellular and intracellular PrP^sc^ was detected by MAb P4 in the zebu and the sheep with scrapie. By contrast, in BSE-affected sheep, PrP^sc^ was detected by MAb P4 in extracellular but not intracellular space (*16*). However, further investigations that use comparative pathology and biologic strain typing would be required to characterize the phenotype of SE in this zebu and the infectious agent in more detail.

Whatever the origin of the disease, this case indicates that zebus are not naturally resistant to SE and, therefore, that B. indicus should be included in programs that monitor transmissible spongiform encephalopathies (TSEs) and in risk assessments in countries where these animals are part of the domestic livestock. Although the potential for this disease to cross the species barrier to other animals and humans is not known, zoos and veterinary services should be aware of the possibility of SEs in such animals so they can subsequently minimize the risk for foodborne SE infections in other animal species (especially Felidae) and humans by removing specified risk materials.
